# Visual and Verbal Processes in Right-Left Confusion: Psychometric and Experimental Approaches

**DOI:** 10.3389/fpsyg.2021.753532

**Published:** 2021-11-10

**Authors:** Ubuka Tagami, Shu Imaizumi

**Affiliations:** ^1^Graduate School of Humanities and Sciences, Ochanomizu University, Tokyo, Japan; ^2^Institute for Education and Human Development, Ochanomizu University, Tokyo, Japan

**Keywords:** right-left confusion, vision, spatial cognition, verbal processing, individual differences

## Abstract

Errors in discriminating right from left, termed right-left confusion, reflect a failure in translating visuospatial perceptions into verbal representation of right or left (i.e., visuo-verbal process). There may also be verbo-visual process, where verbal cues are translated into visual representations of space. To quantify these two processes underlying right-left confusion, Study 1 investigated the factor structure of the Right-Left Confusability Scale, which assesses daily experiences of right-left confusion. Exploratory factor analysis suggested that these two processes and another factor reflecting mental rotation underlie right-left confusion. Study 2 examined correlations between the (sub)scale scores and performance on orientation judgment tasks reflecting visuo-verbal and verbo-visual processes. Overall, self-reported measures were not associated with the behavioral performances presumably reflecting the two processes. These results suggest that the cognitive mechanisms underlying right-left confusion can be classified into visuo-verbal and verbo-visual processes and mental rotation, although their psychometric and behavioral indices might be distinct. Further studies may develop better assessments of right-left confusion reflecting these processes.

## Introduction

Euclidean space has three axes (i.e., up-down, front-back, and right-left). We can make errors in discrimination and identification of right and left in particular. For example, one may misjudge the right or left side of a person’s body (parts), which can even cause medical and traffic accidents (Gormley et al., 2008). Misjudgment of right-left discrimination is called right-left confusion (Wolf, 1973; Hirnstein et al., 2009). In contrast, we rarely misjudge up and down or front and back in space (Corballis and Beale, 1976; Vingerhoets and Sarrechia, 2009). Right-left confusion could reflect the symmetry of our mental right-left axis (Farrell, 1979). Since gravity goes down, upper and lower spaces have robustly distinct identities in our mind. Thus, our mental up-down axis is asymmetric and easily distinguishable, as is the front-back axis because it is based on the innate structure of our body (e.g., the eyes look forward). As for the right-left axis, there is no physical constraint to absolutely determine right and left in space. Moreover, most people have right and left limbs, eyes, and so on, and are able to choose, for example, which limb they will move. In this sense, right and left in space could be relatively symmetric and less distinct, which may be the source in our cognitive system of right-left confusion.

The goal of this study is to elucidate the cognitive mechanisms of right-left confusion. Discrimination between right and left involves both visual and verbal functions (Farrell, 1979; Hirnstein et al., 2011), which could be sequentially connected. Specifically, we perceive and encode visual information (e.g., objects whose right or left is to be judged), and then verbally identify its spatial attributes (e.g., “the object on the right”). This assumed process can underlie everyday situations such as the visual acuity test, where one observes a Landolt C and verbally reports its direction. This can be referred as the visuo-verbal process. On the other hand, the opposite verbo-visual process could be assumed. For example, when you are instructed to turn *to your left*, you might first interpret the verbal information (i.e., instruction) and then translate it into the visual representation of space. This could be referred to as the verbo-visual process. We hypothesized that errors in the visuo-verbal and verbo-visual processes underlie right-left confusion. Individuals can make errors in translating visuospatial information into verbal spatial information, and vice versa. For example, one may not be able to verbally respond immediately to a visually presented direction (e.g., Landolt C) and to move their right or left hand immediately in response to a verbal instruction (Tanioka and Yamashita, 2007).

Proneness to right-left confusion has been studied by behavioral experiments (Farrell, 1979) as well as self-reported questionnaires (Hannay et al., 1990; Jordan et al., 2006). A Japanese self-report measure of right-left confusion, the Right-Left Confusability Scale (RLCS), developed by Tanioka and Yamashita (2007) presented adequate internal consistency (Cronbach’s alpha = 0.83) and test–retest reliability (ICC = 0.81) based on an undergraduate sample (Yamashita, 2013). The RLCS includes items asking for agreement on everyday experiences related to right-left confusion. A higher total RLCS score indicates more difficulty in right-left discrimination (Yamashita, 2013). It is assumed that the RLCS consists of one factor, although factor analysis of RLCS is yet to be performed even by the original developers (Tanioka and Yamashita, 2007; Yamashita, 2013). Thus, the factor structure of the RLCS remains unclear. Therefore, Study 1 in the present study aimed to determine the factor structure of the RLCS and, more importantly, to psychometrically examine whether visuo-verbal and verbo-visual processes underlie right-left confusion.

Study 2 aimed to examine whether the self-reported measure of right-left confusion is associated with behavioral measures (e.g., reaction time for right-left judgment). Indeed, significant correlations have been found between self-reports of one’s own ability to discriminate right from left and behavioral performance measured by the Bergen right-left discrimination test (Gormley et al., 2008) and the Money Road-Map Test (Yamashita, 2013). In the Bergen right-left discrimination test, participants are required to judge the right or left hand of a human figure viewed from his or her front and back (Ofte and Hugdahl, 2002). In the Money Road-Map Test, participants trace a route on a two-dimensional city map while indicating whether a right or left turn is required at each corner (Money et al., 1965). These tests assess the capacity of right-left discrimination based on visual but not verbal instructions, and thus do not examine differences in the assumed visuo-verbal and verbo-visual processes underlying right-left confusion. Therefore, the present study, following a previous study (Farrell, 1979), employed two tasks in which participants orally and manually respond to non-verbal and verbal directional cues, respectively. These tasks were assumed to involve visuo-verbal and verbo-visual processes underlying right-left discrimination and its confusion.

We aimed to examine whether right-left confusion is based on visuo-verbal and verbo-visual processes and to investigate associations between self-reported and behavioral signatures of the two processes in right-left confusion. Study 1 examined whether the RLCS had visuo-verbal and verbo-visual factors underlying right-left confusion. Study 2 employed the RLCS and two experimental tasks to test the following hypotheses: if the self-reported measure correlates with behavioral signatures, then those individuals with higher scores for the visuo-verbal factor of RLCS show slower verbal responses to non-verbal right or left cues because of difficulty in translating visually oriented spatial cues into verbal representation of the space. Furthermore, those with higher scores for the verbo-visual factor display slower manual (non-verbal) response to a verbal right or left cue because of difficulty in translating verbal spatial cues into visual representation of the space.

## Study 1

### Methods

#### Participants

A total of 115 Japanese female undergraduates (mean age of 19.5 years, *SD* = 1.1) participated. Men are less prone to right-left confusion than women (Gormley et al., 2008; McKinley et al., 2015). Thus, only women were recruited to easily observe right-left confusion and investigate visuo-verbal and verbo-visual processes of right-left confusion. All participants reported no history of neurological and psychiatric illness. We analyzed data from the 98 participants who reported they were right-handed to control potentially confounding effect of handedness (Hannay et al., 1990; Constant and Mellet, 2018). Written informed consent was obtained from each participant. The Ethics Committee of Ochanomizu University approved this study (approval number: 2019-174).

#### Measures and Procedure

The RLCS (Tanioka and Yamashita, 2007) comprises nine items asking daily experiences of difficulties in right-left discrimination (Table 1). Participants responded using a 5-point Likert scale (1: *Strongly disagree*; 5: *Strongly agree*). They also answered whether they had a driver license, as Items 8 and 9 ask about car driving. If they did not have the license, they could skip these items. Participants completed the questionnaire and reported their demographic information in introductory psychology classes by a paper-and-pencil method.

**TABLE 1:** Factor structure of the Right-Left Confusability Scale (*n* = 98).

		***M***	***SD***	**Factor loading**			**Communality**
				**Factor 1: verbo-visual**	**Factor 2: mental rotation**	**Factor 3: visuo-verbal**	
1.	I cannot immediately move my right or left hand in response to an instruction by another person.	1.94	0.96	1.05	–0.12	–0.03	0.94
2.	I cannot immediately turn to the right or left in response to instruction by another person.	1.95	1.00	0.87	–0.10	0.08	0.71
5.	I cannot immediately judge the right or left of my body in the mirror.	2.82	1.33	–0.32	0.94	0.03	0.63
3.	I cannot immediately judge the right or left hand and body part of a person in front of me.	2.65	1.30	0.33	0.55	–0.21	0.57
4.	When I am told “your right” and “your left,” I cannot immediately identify the correct direction.	2.51	1.27	0.20	0.51	0.04	0.44
6.	When I am a passenger in a car, I cannot immediately instruct the driver to turn right or left.	2.10	1.19	0.25	0.48	0.14	0.51
7.	In the visual acuity test, I cannot immediately report whether the Landolt C faces the right or left.	1.79	1.13	0.05	0.02	0.98	1.00

	Correlation with Factor 1				0.68	0.32	
	Correlation with Factor 2					0.29	

*Items are sorted by factor loading in descending order. Factor 1 consists of items 1 and 2, Factor 2 consists of items 3–6, and Factor 3 consists of item 7.*

#### Data Analysis

We conducted an exploratory factor analysis using the maximum likelihood method with promax rotation on RLCS with jamovi 1.2.9 (Jamovi Project, 2020). The results of a Bartlett’s test and Kaiser–Meyer–Olkin (KMO) measure demonstrate that our sampling is appropriate for the exploratory factor analysis. The number of factors were determined based on either parallel analysis or scree plots, which had better model-fit indices: root mean square error of approximation (RMSEA), Tucker–Lewis Index (TLI), and Chi-square test, which are useful measures to determine how sufficient the model was for the data (Schmitt, 2011; Xia and Yang, 2019). The extracted factors were named based on the authors’ interpretations to ensure that the factor name did not include words used in the items (Thompson and Daniel, 1996).

### Results and Discussion

Items 8 and 9 were excluded from analysis because 89 participants without a driver’s license skipped these items. Factor analysis was valid, as suggested by Bartlett’s test [χ^2^(21) = 280.50, *p* < 0.001] and a KMO measure of 0.78. Parallel analysis suggested two factors accounting for 55.6% of the total variance, which showed fit indices, RMSEA = 0.084, TLI = 0.94, and χ^2^(8) = 13.56 (*p* = 0.094). In contrast, visual inspection of scree plots suggested three factors that showed better fit indices: RMSEA < 0.001, TLI = 1.08, and χ^2^(3) = 0.16 (*p* = 0.984). Therefore, we determined three factors for the RLCS. Factors 1–3 had eigenvalues of 3.00, 0.51, and 0.17, respectively, and explained 30.3, 23.3, and 14.9% of the variance, respectively. Table 1 shows the factor loadings on each item and the correlations between factors.

Factor 1 comprises Items 1 and 2 (Cronbach’s alpha = 0.90), which describe situations where there is difficulty in raising one’s right or left hand and turning right or left in reaction to a verbal instruction. In these situations, there should be difficulty encoding verbal cues representing space into visual spatial representations. Factor 1 was interpreted as the verbo-visual factor. Factor 2 comprises Items 5, 3, 4, and 6 (Cronbach’s alpha = 0.78). Highly loaded items 5 and 3 describe situations where there is difficulty in identifying the right or left side of one’s own body in a mirror or of another person. In such a situation, for example, his/her right hand appears on one’s (relative) left side. Visual perspective taking through mental rotation, which is the ability to mentally rotate imagined objects in two- or three-dimension without the actual objects or their rotation, helps to solve the discrepancy (Zacks, 2008). Empirical studies suggest that we judge the right or left in the allocentric frame (e.g., the facing person’s hands) based on mental rotation of our own perspective in the egocentric frame to the allocentric frame (Harris et al., 2002; Auer et al., 2008). Factor 2 was thus interpreted as the mental rotation factor. Factor 3 comprised Item 7, which describes a difficulty in verbally reporting the visually identified right or left (i.e., Landolt visual acuity test). This situation suggests right-left confusion in a process where visual representation is translated into verbal information and its vocalization. Factor 3 was interpreted as the visuo-verbal factor.

These results suggest that, as hypothesized, there may be verbo-visual and visuo-verbal processes (Factors 1 and 3) underlying right-left discrimination and its confusion, as measured by self-reports of everyday experiences. Nevertheless, the visuo-verbal factor (Factor 3) has only one item. To better support our interpretation, future studies could revise the RLCS by including more items assessing the visuo-verbal process. According to previous studies suggesting that right-left discrimination requires higher order functions such as mental rotation as well as visual and verbal capacities (Benton and Sivan, 1993; Jordan et al., 2006), we identified Factor 2, which may be related to mental rotation. In the mental rotation factor, Items 3, 5, and 6 could also reflect the visuo-verbal process (e.g., the mental mirrored image is translated into verbal labeling of right or left), while Item 4 reflects the verbo-visual process (e.g., the instruction “your right” is translated into a visuospatial representation of one’s right). Thus, the mental rotation factor may reflect not only mental rotation employed for right-left discrimination but also partially overlap with the visuo-verbal and verbo-visual processes. In addition, our factor analysis revealed that the variance of the RLCS score was best explained by the verbo-visual factor (Factor 1). People may experience right-left confusion especially when they translate verbal cues of spatial information into visuospatial information. The ability to convert verbal to visual, rather than visual to verbal, spatial information could be influential in right-left confusion. In contrast, the subscale score of the mental rotation factor (Factor 2, *M* = 2.52, *SD* = 0.99) was higher than that of the verbo-visual factor [Factor 1, *M* = 1.94, *SD* = 0.93, *t*(97) = 6.28, *p* < 0.001, Cohen’s *dz* = 0.63], suggesting that the female participants had difficulty in right-left discrimination, particularly when they were required to employ mental rotation. This may reflect a tendency in women to be confused while performing mental rotation tasks (Peters et al., 2006), although a potential contribution of the verbo-visual process is retained because sex differences in right-left confusion are not explained by differences in mental rotation alone (Ocklenburg et al., 2011). Taken together, Study 1, employing a self-reported measure, suggested visuo-verbal and verbo-visual processes underlying right-left confusion.

## Study 2

Study 2 aimed to examine whether self-reported measures correlate with behavioral measures in experimental tasks involving two processes in right-left confusion. Our alternative hypothesis was that scores of the RLCS’s visuo-verbal factor positively correlate with reaction time for verbal responses to visual right or left cues, whereas scores of the verbo-visual factor positively correlate with reaction time for manual responses to verbal right or left cues.

### Methods

#### Participants

Forty of the participants whose data were analyzed in Study 1 participated in Study 2 about a month later (mean age of 19.9 years, *SD* = 1.2). All had normal or corrected-to-normal vision.

#### Stimuli and Apparatus

Stimuli were arrows and Japanese kanji characters indicating spatial orientation. They were presented against a gray background on a 11-inch LCD monitor (MacBook Air, Apple). Participants observed the monitor from a distance of approximately 57 cm without a chin rest. Stimuli subtended approximately 10.2° × 10.2° in visual angle. The kanji were displayed in Kozuka Gothic Pro font. Participants responded using a built-in keyboard. Stimulus presentation and response collection were controlled by PsychoPy 1.85.0 (Peirce et al., 2019) running on macOS 10.12.3.

#### Procedure

**FIGURE 1:**
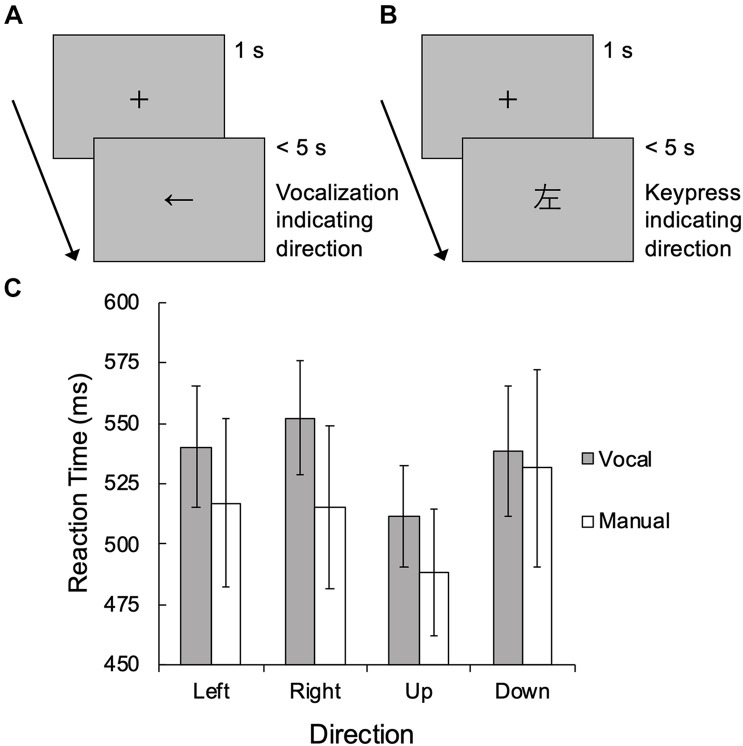
Schematic illustration of **(A)** the vocal task and **(B)** the manual task in Study 2. **(C)** Mean reaction time in Study 2. Error bars denote 95% confidence intervals.

We conducted the vocal and manual tasks following Farrell (1979). We assumed that the vocal task (Figure 1A) involves the visuo-verbal process in spatial cognition, where visuospatial information is translated into verbal spatial information, whereas the manual task (Figure 1B) involves the verbo-visual process where verbal spatial information is translated into visuospatial information.

At the beginning of each trial in the two tasks, a fixation cross was presented for 1 s, then an arrow or kanji character was presented at the center of the monitor until response or 5 s had passed without response (Figures 1A,B). In the vocal task, an arrow directing upward, downward, leftward, or rightward was presented. Participants orally responded with the arrow direction (i.e., 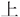 *ue* “up,” 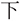 *shita* “down,” 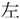 *hidari* “left,” or 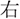 *migi* “right” in Japanese) and simultaneously pressed a key to record their reaction time. Keys were labeled with kanji indicating directions (i.e., the I, M, J, and L keys were labeled 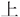, 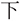, 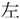, and 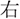, respectively). In the manual task, a kanji character representing an upward, downward, leftward, or rightward direction (i.e., 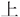, 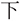, 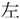, or 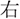, respectively) was presented. Participants responded by pressing a key labeled with an arrow (i.e., the R, C, D, and G keys were labeled ↑, ↓, ←, and →, respectively). In both tasks, participants were asked to respond as quickly as possible with their right hand.

Participants performed 8 practice trials and subsequently 15 trials for each of the up, down, left, and right conditions in a randomized order. Thus, 60 trials were performed for each of the vocal and manual tasks. The tasks were performed in separate blocks. The order of the blocks was counterbalanced. Between blocks, participants performed 10 trials of subtractions of six from two-digit figures and subsequently took a rest for a few minutes to prevent a carry-over effect on the following task.

### Results and Discussion

#### Reaction Time

Two participants were excluded from analysis because their mean reaction time across the left and right conditions exceeded the third quartile plus 1.5 interquartile range. Repeated-measures analysis of variance with Direction (left, right, up, and down) and Task (vocal and manual) as within-participant factors was performed on reaction time (Figure 1C). We found a significant main effect of Direction [*F*(3, 111) = 9.82, *p* < 0.001, $\eta_{p}^{2}=0.210$] and its interaction with Task [*F*(3, 111) = 3.31, *p* = 0.023, $\eta_{p}^{2}=0.082$]. The main effect of Task was not significant [*F*(1, 37) = 3.72, *p* = 0.061, $\eta_{p}^{2}=0.091$], while the simple main effect of Direction was significant for both tasks [vocal: *F*(3, 111) = 6.41, *p* < 0.001; manual: *F*(3, 111) = 10.13, *p* < 0.001]. *Post hoc* comparisons with Bonferroni correction revealed that reaction times under the up condition were significantly faster than under the other conditions (*p*s < 0.001), and there was no difference between the left and right conditions (*p* = 0.999). Slower reaction times in the vocal task were found for the right [*F*(1, 37) = 7.86, *p* = 0.008] and up [*F*(1, 37) = 4.37, *p* = 0.044]) conditions, but not for the left [*F*(1, 37) = 3.38, *p* = 0.074] and down [*F*(1, 37) = 0.22, *p* = 0.640] conditions.

We calculated mean reaction time across the right and left conditions as a raw index of the degree of right-left confusion for each participant. Mean reaction time across the up and down conditions served as a baseline likely to reflect motor-response performance but not the degree of right-left confusion. Finally, the ratio of the reaction time under the right and left conditions to that under the up and down conditions was termed the right-left response delay and served as a standardized behavioral measure of right-left confusion (vocal task; *M* = 1.04, *SD* = 0.08 and manual task; *M* = 1.01, *SD* = 0.07). We assumed that a larger right-left response delay indicates a slower response in right-or-left judgments and thus stronger right-left confusion. Although not the main focus of this study, we found a moderate positive correlation between the right-left response delay in the two tasks (Spearman’s rho = 0.55, *p* < 0.001), suggesting that visuo-verbal and verbo-visual processes can similarly modulate right-left discrimination and are associated within the individual. Moreover, our exploratory analysis revealed that the right-left response delay in the manual task was significantly larger than in the vocal task [*t*(37) = 2.71, *p* = 0.010, Cohen’s *dz* = 0.44], suggesting that people are more likely to experience right-left confusion when they encode verbal spatial cues into a visual representation.

#### Correlations Between the Behavioral and Self-Report Measures

We analyzed the Pearson’s and Spearman’s rank correlations between the right-left response delay and the RLCS completed in Study 1 (Table 2). We examined the rank correlation whenever either variable intended for correlation was non-normally distributed based on the Shapiro–Wilk test. The mean item scores of the RLCS served as (sub)scale scores (total; *M* = 2.49, *SD* = 0.85, verbo-visual; *M* = 2.16, *SD* = 1.01, mental rotation; *M* = 2.80, *SD* = 0.96, and visuo-verbal; *M* = 1.95, *SD* = 1.31), where a higher score indicates stronger right-left confusion. Contrary to our hypothesis, there were no significant correlations between the right-left response delay in the vocal task and the visuo-verbal score. The right-left response delay in the manual task was significantly positively correlated with the total RLCS score (*r* = 0.36, *p* = 0.028) and the mental rotation score (*r* = 0.35, *p* = 0.032); however, again, contrary to the hypothesis, it was not correlated with the verbo-visual score.

**TABLE 2:** Correlation coefficients between the Right-Left Confusability Scale and the right-left response delay in the vocal and manual tasks.

	Right-Left Confusability Scale			
	Total	**Verbo-visual**	Mental rotation	**Visuo-verbal**
**Vocal task**				
Pearson	0.21 (0.210)	0.11 (0.527)	0.19 (0.267)	0.24 (0.156)
Spearman	0.06 (0.721)	−0.03 (0.855)	0.03 (0.880)	0.21 (0.204)
Manual task				
Pearson	0.36 (0.028)*	0.21 (0.200)	0.35 (0.032)*	0.27 (0.107)
Spearman	0.27 (0.099)	0.20 (0.235)	0.24 (0.155)	0.22 (0.181)

p-Values in parentheses, *p < 0.05; *n* = 38. Non-normally distributed variables are bolded (Shapiro–Wilk test, vocal task; *p* = 0.022, verbo-visual and visuo-verbal; *p* < 0.001).

## General Discussion

We perceive visual information representing spatial direction and translate it into verbal information in right-left discrimination. Contrariwise, verbal cues for directions are also processed into a visual representation of space. We hypothesized that these visuo-verbal and verbo-visual processes may underlie right-left discrimination, and when they fail, we experience right-left confusion. This hypothesis was examined in Study 1, which conducted exploratory factor analysis of a self-reported measure of right-left confusion (the RLCS). As hypothesized, the results suggested that everyday situations where we (fail to) discriminate right from left could be classified as reflecting visuo-verbal and verbo-visual processes. Moreover, mental rotation was suggested as another factor of right-left confusion.

In Study 2, participants performed the right-left judgment tasks, presumably involving visuo-verbal and verbo-visual processes. We tested whether the behavioral measures of right-left confusion were correlated with the RLCS. Contrary to our prediction, the scores of the visuo-verbal and verbo-visual factors of the RLCS were not correlated with the behavioral measures in the tasks. These results suggest that there is a gap between self-reported proneness to right-left confusion and cognitive capacities to discriminate right from left. On the other hand, the right-left response delay in the manual task where participants responded by pressing a key was correlated with the total RLCS score in Study 2. These results were consistent with studies that have shown correlations between self-reported measures and behavioral performance in visuospatial tasks requiring manual responses (Gormley et al., 2008; Yamashita, 2013). The right-left response delay in the manual task was also correlated with the mental rotation score of the RLCS, suggesting that multiple cognitive processes could be assumed in investigations of right-left confusion. However, it remains unclear why performance in the vocal task did not correlate with the self-reported measure. Moreover, as the verbo-visual and visuo-verbal factors of the RLCS include only one or two items, the reliability and validity of the subscales could be improved.

Our study has four limitations. First, our samples included only women. Yamashita (2013) reported that women judge themselves as having more difficulty in right-left discrimination than men, although such sex difference was not found for accuracy in the Money Road-Map Test. To generalize our results, future studies including men and women are needed. Second, we excluded two items of the RLCS from factor analysis due to a large number of missing responses (see also Results of Study 1), potentially biasing the results of factor structure and the relationships with behavioral measures. Third, the procedure of the vocal task in Study 2 can be considered as a limitation. Participants orally responded with the arrow direction and virtually simultaneously pressed a key. Although the reaction time was defined as the time of keypress, this procedure might not have recorded the actual time of vocal response due to temporal lags between vocalization and keypress movement. Although similar effects of stimulus direction were found for both the vocal and manual tasks (Figure 1C), the generally delayed responses in the vocal task might be attributable to this response procedure. Finally, we did not conduct the behavioral task to assess mental rotation (Factor 2 of the RLCS) because that was not main focus of our study. However, future studies should examine the whole aspect of the mechanism of right-left confusion including visuo-verbal and verbo-visual processes and mental rotation.

Future studies should consider cognitive domains involved in right-left discrimination and their dysfunction resulting in right-left confusion. Both visuo-verbal and verbo-visual processes will require visuospatial and linguistic abilities. Generally, people have right-hemispheric spatial dominance and left-hemispheric language dominance, but left-handed or ambidextrous people may have atypical lateralization (Szaflarski et al., 2002). Our studies included only right-handed participants as previous studies suggested that left-handed people had advantage (Constant and Mellet, 2018) or disadvantage (Hannay et al., 1990) in right-left discrimination compared to right-handers. We may be able to reveal whether and how handedness affects right-left discrimination by considering the two processes and cortical lateralization. Sex differences may also be observed in visuospatial and linguistic abilities. Such differences in right-left confusion are not explained by those in mental rotation (Ocklenburg et al., 2011) but are associated with prefrontal cortical excitability (Hjelmervik et al., 2015) and cortical lateralization. Hirnstein et al. (2009) reported that women with right ear advantage in dichotic listening (i.e., left-hemispheric advantage) showed greater right-left confusion than in such men. Importantly, there was no such sex difference in less lateralized people. A potential factor of individual differences in the ability of right-left discrimination may be the cortical lateralization; stronger lateralization may bias the exchange or translation of visual and verbal spatial information between hemispheres. Further studies on visuo-verbal and verbo-visual processes in right-left confusion should be performed using broader sample to examine potential (interactive) effects of cortical lateralization, handedness, and sex.

## Conclusion

In conclusion, we suggested that cognitive mechanisms underlying right-left confusion could be classified into visuo-verbal and verbo-visual processes and mental rotation based on a self-reported measure. However, we did not find significant associations between the self-reported and behavioral measures for verbo-visual and visuo-verbal processes. There is room for improvement in the psychometric and behavioral assessments of right-left confusion.

## Data Availability Statement

The datasets analyzed for this study can be found at the Open Science Framework (https://osf.io/4y7e5/).

## Ethics Statement

This study was reviewed and approved by the Ethics Committee of Ochanomizu University. The participants provided their written informed consent to participate in this study.

## Author Contributions

UT conceived the study and performed the experiments. UT and SI analyzed the data and wrote the manuscript. Both authors approved the final version of the manuscript.

## Conflict of Interest

The authors declare that the research was conducted in the absence of any commercial or financial relationships that could be construed as a potential conflict of interest.

## Publisher’s Note

All claims expressed in this article are solely those of the authors and do not necessarily represent those of their affiliated organizations, or those of the publisher, the editors and the reviewers. Any product that may be evaluated in this article, or claim that may be made by its manufacturer, is not guaranteed or endorsed by the publisher.
